# Synthesis and Biological Activities of Dehydrodiisoeugenol: A Review

**DOI:** 10.3390/ph15111351

**Published:** 2022-10-31

**Authors:** Beatriz Godínez-Chaparro, Salud Pérez-Gutiérrez, Julia Pérez-Ramos, Ivo Heyerdahl-Viau, Liliana Hernández-Vázquez

**Affiliations:** Departamento de Sistemas Biológicos, Universidad Autónoma Metropolitana Unidad Xochimilco, Calzada del Hueso No. 1100, Mexico City 04960, Mexico

**Keywords:** dehydrodiisoeugenol, licarin A, synthesis, biocatalysts, biological activities

## Abstract

Dehydrodiisoeugenol (DHIE) is a neolignan found in more than 17 plant species, including herbs, fruit, and root. DHIE was, for the first time, isolated from *Myristica fragrans* bark in 1973. Since then, many methodologies have been used for the obtention of DHIE, including classical chemistry synthesis using metal catalysts and biocatalytic synthesis; employing horseradish peroxidase; peroxidase from *Cocos nucifera*; laccase; culture cells of plants; and microorganisms. Increasing evidence has indicated that DHIE has a wide range of biological activities: anti-inflammatory, anti-oxidant, anti-cancerogenic, and anti-microbial properties. However, evidence in vivo and in human beings is still lacking to support the usefulness potential of DHIE as a therapeutic agent. This study’s review was created by searching for relevant DHIE material on websites such as Google Scholar, PubMed, SciFinder, Scholar, Science Direct, and others. This reviews the current state of knowledge regarding the different synthetical routes and biological applications of DHIE.

## 1. Introduction

**Figure 1:**
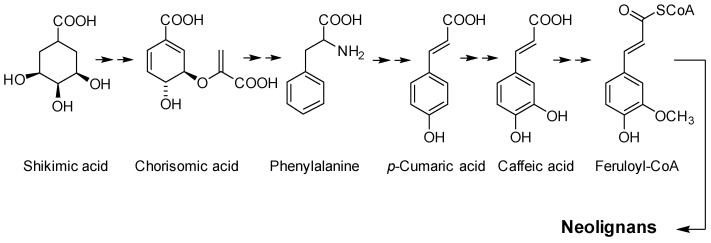
Shikimic acid pathway neolignans biosynthesis.

Lignans and neolignans are secondary metabolites found in plants and animals. These compounds play an essential role in plants’ defenses against microorganisms [1] and phytophagous animals and insects [2]. Neolignans are dimers of phenylpropane originating from the shikimic acid pathway [3], and deamination of phenylalanine leads to caffeic acid (Figure 1). Lignans are dimers of phenylpropanoid units linked via their β-carbon atoms [4]. When the phenylpropanoid units are coupled via other linkages, they are named neolignans [5]. Many neolignans show different biological activities, such as cytotoxicity, anti-oxidant, anti-inflammatory, and anti-parasitic, among others [6].

**Figure 2:**
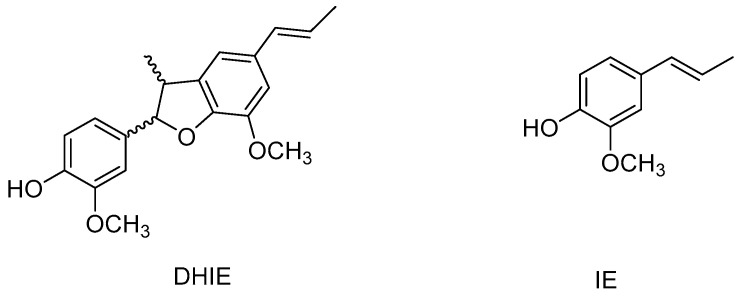
Structure of DHIE and IE.

One of these neolignans is dehydrodiisoeugenol (DHIE). In 1973, this compound was isolated for the first time from *Myristica fragrans* bark [7]; later, DHIE was separated from the wood of *Licaria aritu* [8]. Nevertheless, DHIE had already been obtained by isoeugenol (IE) oxidation, and its structure was determined before it was isolated from a natural source (Figure 2) [7]. Several studies have demonstrated that DHIE shows a wide range of biological actions such as anti-bacterial [9], antiprotozoal [10], anticancer [11], anti-oxidant [12], and anti-inflammatory [12].

This study’s review was created by searching for relevant DHIE material on websites such as Google Scholar, PubMed, SciFinder Scholar, Science Direct, and others. This study reviews the current state of knowledge regarding the different via produced synthetically and the multidirectional action and application of DHIE.

## 2. Characteristics of DHIE

DHIE (C_20_H_22_O_4_), also known as licarin A, is a neolignan belonging to the phenols group. It is commonly obtained from different plant families such as *Aristolochiaceae*, *Lauraceae*, *Magnoliaceae*, and *Piperaceae*, although it is well known that the compound can be found in various concentrations (ranging from 0.08–0.53 mg/g plant) depending on the species (Table 1). DHIE is a colourless crystal with a melting point of 132–133 °C and an intense aroma [7]. This compound is sparingly soluble in water and well soluble in organic solvents such as ethyl acetate and dichloromethane. There are two ways to synthesize DHIE: classical chemistry and biocatalysis.

## 3. Classical Synthesis of DHIE

**Figure 3:**
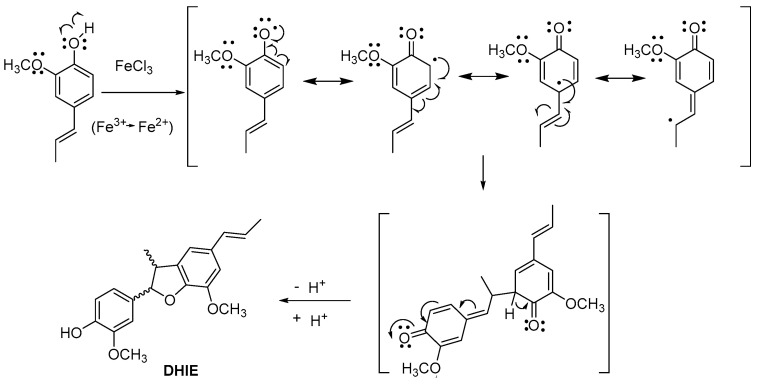
Reaction mechanism for the oxidative dimerization of isoeugenol.

DHIE is isolated from different plant species with yield low. Therefore, the synthesis of DHIE is an alternative for obtaining significant quantities. In this sense, classical chemical synthesis from IE produces the best yields. Moreover, various catalysts have been used for this purpose; these methodologies are described in the following. In 1950, Leopold synthesized DHIE by IE, using ethanol-water as the solvent and ferric chloride as the catalyst and obtaining a yield of 30% [34]. The reaction mechanism of oxidative dimerization of isoeugenol is shown in Figure 3. This method was later modified by Levita et al., decreasing the amount of catalyst and yield [35].

Liu et al. used the radical dimerization reaction of IE, employing Ag_2_O as the catalyst, in toluene and acetone at room temperature to yield 40% of DHIE [36]. Chen et al. developed a new method for the diastereoselective oxidative dimerization of isoeugenol under different reaction conditions. For example, when they used 1.5 mol of cerium ammonium nitrate (CAN) in tetrahydrofuran and had a 0.5 h reaction time, the yield of DHIE was 81% [37]. Juhász et al. developed a method using isoeugenol and iodobenzene diacetate (IDA) in dichloromethane at room temperature and obtaining a yield of 35% [38]. Table 2 compares the reaction conditions for radical dimerization of IE.

## 4. Biocatalytic Synthesis of DHIE

The synthesis of DHIE by biocatalysis employed different sources for enzymes as catalysts in mild and environmentally friendly reaction conditions and produced a yield of range 20 to 98%. This method did not use metal catalysts; for these reasons, biocatalysis is an excellent option to obtain DHIE. The obtaining of DHIE proceeds via oxidative processes catalyzed by enzymes such as peroxidase and laccase, which convert the phenol to phenoxy radical by an electron abstraction followed by carbon-carbon and carbon-oxygen bond formation [39].

### 4.1. Horseradish Peroxidase for Synthesis of DHIE

Krawczyk et al. synthetized DHIE for the first time from IE using horseradish peroxidase (HRP) and hydrogen peroxide, with a 25 h of reaction time and a pH of 6, which resulted in a 22% yield in methanol-buffer [40]. Chioccara et al. studied the effect of methanol ratio in the reaction mixture and pH for the DHIE synthesis, finding that the highest yield was obtained with 10% methanol at pH 3 (Table 3) [39]. Nascimento et al. used HPR and H_2_O_2_ to transform isoeugenol to DHIE with a 99% yield. They obtained both enantiomers; subsequently, they were transformed to the α-methoxy-α-trifluoromethylphenylacetate derivatives, and these diastereomers were separated by HPLC, and they were transformed to (+)-DHIE and (−)-DHIE [41].

Bortolomeazzi et al. used the HPR and H_2_O_2_ in the synthesis of DHIE from IE (mixture of E and Z isomers) in methanol-citrate-phosphate buffers to obtain a crystalline product (yield 25%) containing four DHIE isomers and [(E)-(±)-trans-dehydrodiisoeugenol] being the most abundant of this mixture (yield 95.7%) [42]. Pereira et al. obtained 98.3% with the HPR-H_2_O_2_ system of (±)-DHIE, and the resolution of enantiomers was then directly performed by HPLC with an analytical ChiralPak AD column [43].

### 4.2. Peroxidase of Cocos nucifera L. for Synthesis of DHIE

The coconut water, obtained from green coconuts (*Cocos nucifera* L.), contains peroxidases [44]. IE is transformed of DHIE using coconut water and H_2_O_2_ with a 55% yield, in this method, only the enantiomer (−)-DHIE was obtained. Authors suggest that this effect is due to the presence of an auxiliary protein in the coconut water [44]. In another study, the use of coconut water as a catalyst yielded 60% of the enantiomer (−)-DHIE [45].

### 4.3. Laccase for Synthesis of DHIE

The pure laccase isolated from *Rhus vernicifera* Stokes (tree) catalyzed the oxidation of IE to give a mixture of dimeric and tetrameric compounds. The main product of this reaction was DHIE, with a 41% yield [46].

### 4.4. Cell Culture of Plants for Synthesis of DHIE from IE

The DHIE can also be obtained from IE, using nine plant cell cultures or calli of *Medicago sativa*, *Phaseolus vulgaris*, *Mamilaria huitzilopochtli*, *Psacalium composite*, *Cucumis melo*, *Prunus serotina*, *Bovardia ternifolia*, *Coriandrum sativum* and *Dacus carota*. In this method, three compounds were observed where DHIE was obtained in higher amounts. The best yield of DHIE (23%) was obtained with *B. ternifolia* [47]. In another study, DHIE was obtained from IE with *B. ternifolia* cultivated under nutritional stress, and H_2_O_2_ with a yield of 77% [48].

### 4.5. Microorganisms for Synthesis of DHIE

(+)-DHIE was obtained from IE biocatalyzed by *Pseudomonas putida* NCIM 2176 cell culture with a yield of 16% [49]. In addition, IE was transformed into the racemic mixture of DHIE by *Bacillus pumulus* [50].

## 5. Synthesis of DHIE Derivatives

**Figure 4:**
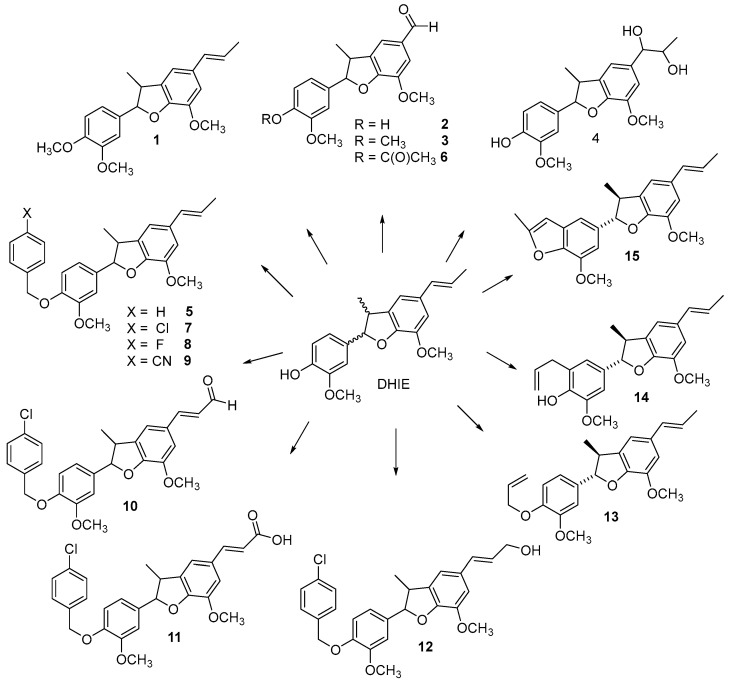
Semi-synthetic derivatives of DHIE.

Different derivatives of DHIE have been obtained by structural modifications in order to improve their biological potential and physiochemical properties. The modifications of DHIE involve the reaction of the propenyl chain and phenolic hydroxyl (Figure 4).

Compound **1** (Figure 4) was obtained from (±)-DHIE using methyl iodide and K_2_CO_3_ in acetone (yield 77%) [51]; or methyl iodine and NaH in tetrahydrofuran (56% yield) [52]; or dimethyl sulphate and K_2_CO_3_ and acetone (yield 86%) [36]; in addition, in another study, instead of K_2_CO_3_, NaOH in ethanol was used [53].

The aldehyde **2** was synthetized from (±)-DHIE, and aldehyde **3** from compound **1** using KIO_4_ and OsO_4_ in water-tetrahydrofuran with a yield of 96% and 90%, respectively [51].

The reaction of DHIE with meta-chloroperoxybenzoic acid in dichloromethane gave compound **4** (yield of 20%) [51]. Juhasz et al. obtained derivative **4** from DHIE with OsO_4_ in dioxane with a yield of 49% [38].

Compound **5** was prepared from DHIE with benzyl bromide (58% yield) [54]. Juhasz et al. obtained derivate **5** from DHIE with benzyl chloride and K_2_CO_3_ in dimethylformamide with a yield of 61% [38].

Compound **6** was synthesized from (±)-DHIE by acetylation with acetic anhydride and pyridine in tetrahydrofuran (18% yield) [52]. Also, compound **6** was obtained with acetyl chloride and triethyl amine (23% yield) [54]. Compounds **7**, **8** and **9** were obtained from (−)-DHIE and benzyl halide with K_2_CO_3_ in dimethylformamide. The aldehyde **10** was synthesized from compound **7** using 2,3-dichloro-5,6-dicyano-1,4-benzoquinone in water with a yield of 87%. The carboxylic acid **11** was obtained from aldehyde **10** and Ag_2_O (yield 41%). The derivative **12** was synthesized from **10** and NaBH_4_ in ethanol with a yield of 70% [54].

The mixture of DHIE and allyl bromide with K_2_CO_3_ in acetone produced derivative **13** (yield 73%). Compound **13** in dimethylformamide was submitted to microwave irradiation producing **14** (yield 53%). The derivative **15** was obtained by the reaction of **14** and I_2_ in ethanol water with a yield of 7% [18].

**Scheme 1:**
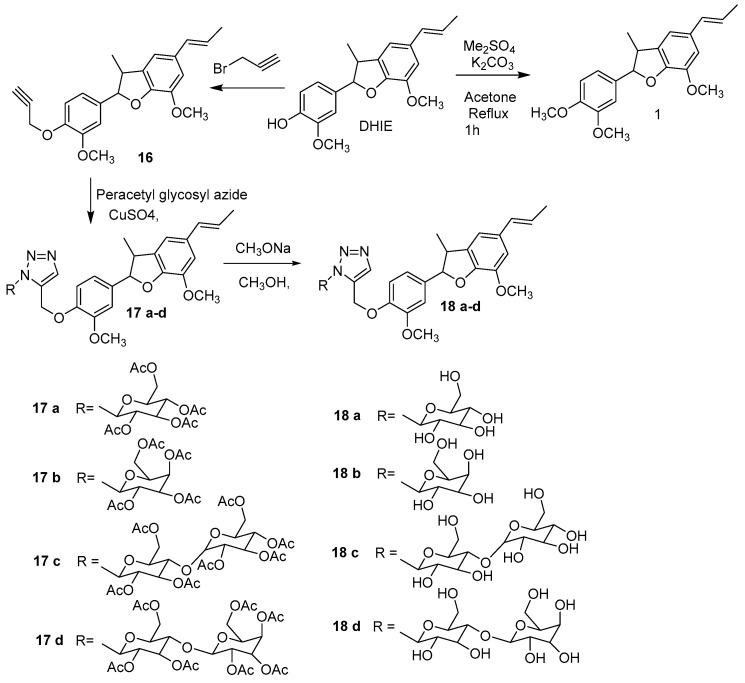
Synthesis of triazoylglycosyl from DHIE.

Triazolylglycosides **17a**–**d** and **18a**–**d** were synthetized using acetylenic-(±)-DHIE (**16**) and acetylated sugar (Scheme 1) [36].

## 6. Biological Activity of DHIE

DHIE (5 mg/kg/21 days, subcutaneous) toxicity studies have shown normal histological architecture of the liver and kidney, and functional biochemical tests were also normal, suggesting that DHIE produced no toxic effect, at least in mice [9]. Given the low toxicity of the DHIE, it is interesting to pay attention to the potential biological properties of this compound.

### 6.1. Anti-Oxidant Effect of DHIE

Oxidative stress is produced by free radicals, which could cause health problems such as cancer, inflammation, and neurodegenerative diseases, among others. Anti-oxidants have the ability to capture free radicals inhibiting oxidative stress and helping in the prevention of these diseases [55]. DHIE has demonstrated anti-oxidant and neuroprotective properties. As an anti-oxidant, DHIE preserves the activities of anti-oxidant enzymes such as superoxide dismutase, glutathione peroxidase, and glutathione reductase in the glutamate-injured neuronal cells [56]. In addition, DHIE increases reactive oxygen species scavenging activity [12,57,58,59] and inhibits the production of nitric oxide [56].

DHIE shows 2,2-diphenyl-1-(2,4,6-trinitrophenyl)hydrazyl (DPPH) radical scavenging activity with the IC_50_ values of 1.312 mM [12], 5.320 mM [58], 0.075 mM [56], and 66.02 µg/mL [59]. Moreover, Hou et al. reported that DHIE shows 12% of scavenging activity [57]. In contrast, Lin et al. reported that DHIE did not exhibit DPPH radical scavenging activities [28]. Taken together, these data suggest that DHIE shows only weak anti-DPPH radical activity [58]. Furthermore, the anti-oxidant activity could be evaluated with a 2,2′-azinobis-(3-ethylbenzothiazoline-6-sulfonic acid) (ABTS) assay. In this context, DHIE showed high ABTS radical scavenging activities-IC_50_ = 8.43 µg/mL [59], IC_50_ = 12.3 µM [28], and ≈60% [57] of the scavenging activity. Also, DHIE showed relatively high hydroxyl radical scavenging activity. However, DHIE has no effect on the superoxide radical [59]. The potential anti-oxidant activity exhibited by DHIE could be explained by its structural proprieties as: (1) DHIE contains an OH group at C(4) that is important for the anti-oxidant activity; (2) DHIE has a methoxy group at C(4) in ring C, and this group reduced the ABTS cation radical-scavenging activity [28].

### 6.2. Anti-Parasitic Activity of DHIE

Neglected tropical diseases (NTD) are a group of diseases that prevail in developing countries and affect more than one billion people. Among them is Chagas disease, leishmaniasis, and schistosomiasis. *Trypanosoma cruzi* is transmitted by insect bites and is responsible for the Chagas disease. Species of Leishmania, including *Leishmania amazonensis*, among many others, are transmitted to humans by the bite of a sand fly, causing leishmaniasis disease. Schistosomiasis is caused by trematode worms of the *Schistosoma genus* [60].

DHIE has been demonstrated to exert trypanocidal activity against *T. cruzi*, inducing swelling of mitochondria and disorganization of mitochondrial cristate, the Golgi complex, and other organelle alterations [10]. Other authors studied the activity of (±)-DHIE and the enantiomers against trypomastigotes of *T. cruzi* [10,18,52,61]; they found that (−)-DHIE displayed the best activity with IC_50_ of 23.46 μg/mL, and (+)-DHIE had a lower effect with IC_50_ value of 87.73 μg/mL, and the racemic mixture showed the lowest activity with IC_50_ of 127.17 μg/mL [43,52].

DHIE inhibited promastigotes of *Leshmania major* with the IC_50_ of 9.59 µg/mL, inducing apoptosis. Also, this compound was active against intracellular amastigotes in murine macrophages with the EC_50_ = 4.71 µg/mL, showing better activity than the meglumine antimoniate (EC_50_ = 216.2 µg/mL) reference drug [45].

DHIE was tested against *Schistosomas mansoni* and killed 100% of the adult worms at 50 µM (EC_50_ = 25.94 µM) without affecting the cell line [17]. The (±)-DHIE displayed the best activity against *S. mansoni* adults (LC_50_ = 53.57 µM); the (−)-DHIE showed low activity (LC_50_ = 91.71 µM); whereas the (+)-DHIE had no activity (LC_50_ = 209.4 μΜ) [43]. In this line, Meleti et al. reported that (±)-DHIE at 200 µg/mL induced 100% of mortality of *S. mansoni* [52]. Moreover, the oral administration of 400 mg/kg of DHIE to mice infected with *S. mansoni* reduced worm burden by 50% and decreased the number of immature eggs by 50–60%, suggesting that DHIE could be used in association with another anti-parasitic as praziquantel to achieve a synergic effect [17].

### 6.3. Anti-Bacterial Activity of DHIE

One of the main problems with bacterial infections is antibiotic resistance, which has increased worldwide. *Mycobacterium* is not the exception, and many species of this genus have developed multidrug resistance (MDR). In recent years, the number of patients with tuberculosis (TB) has increased, particularly, in those patients with compromised immunological systems [62].

DHIE has been effective against *Mycobacterium* strains [9,13,54]. Moreover, DHIE displayed antimycobacterial activity against *Mycobacterium abscesses* (MIC = 9.76 μg/mL), *Mycobacterium fortuitum* (39.06 μg/mL), and *Mycobacterium massiliense* (39.06 μg/mL). Inhibition of biofilm formation of these strains was also tested, but no significant activity was observed. Also, it was found that DHIE had activity against non-tuberculosis *Mycobacterium* strains (MIC = 3.12–6.25 μg/mL). Moreover, DHIE has been shown to be effective against four multiresistant *Mycobacterium* strains and 12 strain clinical isolation (MIC = 3.12–25 µg/mL) [13]. (−)-DHIE was tested in a Tuberculosis animal model, inducing disease with *M. tuberculosis* H37Rv or an MDR, where the dose of 5 mg/kg decreased the pulmonary bacillary burdens at day 30 of treatment, and a significant pneumonia reduction at days 30 and 60 of treatment [9]. However, this compound showed no activity in some bacteria, such as *Escherichia coli*, *Pseudomonas fluorescens*, and *Listeria monocytogenes* [63].

### 6.4. Anticancer Activity of DHIE

Cancer is a group of diseases that is characterized by uncontrolled cell growth, and it has the potential to invade or spread to other organs or tissue of the body. The cancer cells evade growth suppressors, resisting cell death and enabling replicative immortality. Cancer is one disease with significant morbidity and mortality over the world; is the second worldwide cause of death (8.97 million deaths) after ischemic heart disease, and it is expected to increase in the next years [64].

The cytotoxic activity of DHIE has been studied on different cancer cell lines, such as leukemia (CEM/ADR5000), breast cancer (MDA-MB-231-pcDNA, MDA-MB-231-BCRP), colorectal cancer (HCT116p53+/+, HCTp53−/−), glioblastoma (U87MG), hepatoblastoma (HepG2), melanoma (BRAF-V600E, MAMEL-80A, SK-MEL28, BRAF-V600E-A2058, BRAF wildtype, MV3, SK-MEL505), rat adenocarcinoma (CC531), mouse melanoma (B1-F1 and B16-F10); the IC_50_ values varied from 4.3 to 22.4 µg/mL. Also, the leukemia cell line (CCRF-CEM) treated with the DHIE cell cycle was analyzed by flow cytometer; the results indicated that this compound induced apoptosis [26].

DHIE inhibited the growth of HCT116 and SW620 (colorectal cancer) cell lines with IC_50_ values of 54.32 and 46.74 µM, respectively. The cell cycle was examined by a flow cytometer. The results indicated that DHIE inhibited the cell growth of both colorectal cancer lines by the induction of cell cycle arrest and induced autophagy in HCT116 and SW620 cell lines [11].

DHIE showed cytotoxic activity against A-549, MCF7, and HCT-15 cell lines; the IC_50_ values were 2.0, 1.6, and 10.0 µM, respectively. This compound inhibited in vitro phospholipase Cγ1 (IC_50_ value = 15.8 µM), which plays an essential role in the proliferation and progression of human cancer [65].

DHIE showed anti-proliferative activity against four non-small cell lung cancer cell lines (A549, NCI-H23, NCI-H520, and NCI-H460) with IC_50_ values of 22.19, 20.03, 30.20, and 35.01 µM, respectively. In NCI-H23 and A549, DHIE induced autophagy and its association with apoptosis [66].

These studies show that DHIE could be a candidate to be a chemo-therapeutic agent and could prevent cancer.

### 6.5. Anti-Inflammatory Effect of DHIE

Inflammation is a set of physiological processes that an organism starts in response to foreign stimuli, including pathogens, such as viruses and bacteria, and inorganic particles [67]. Inflammation is a response to diverse diseases ranging from trauma and infection to immune-mediated disease and neoplasia. As such, inflammation can be a nonspecific finding but remains a valuable indicator or pathology that can itself lead to disease whether left unchecked [68]. In this sense, recent investigations have shown that inflammation is a major factor in the progression of various chronic diseases/disorders, including diabetes, cancer, cardiovascular diseases, eye disorders, arthritis, obesity, autoimmune diseases, and inflammatory bowel disease [69].

Some evidence has demonstrated that DHIE has anti-inflammatory properties. In this line, Murakami et al. and Fujisawa et al. found that DHIE inhibits the expression of COX-2, but not COX-1, in RAW264.7 murine macrophages and RBL-2H3 cells when stimulated with lipopolysaccharide (LPS) [12,58] and dinitrophenyl-human serum albumin (DNP-HAS), respectively. Moreover, the DHIE decreases the levels of prostaglandin D2 (PGD2) in DNP-HAS-stimulated RBL-2H3 cells [70]. These data suggest that DHIE may be an inhibitor of COX-2 gene expression and, as a result, may inhibit the production of PGD2.

(±)-DHIE decreases the expression of p65 NF-κB in DNP-HAS-stimulated RBL-2H3 cells [70] and inhibits NF-κB binding in RAW264.7 cells [58]. Moreover, it is widely known that LPS can stimulate Iκ-B phosphorylation and degradation in macrophage cell cultures; however, in cell treatment with DHIE, the Iκ-B-α phosphorylation and degradation are inhibited [58]. These data strongly suggest that DHIE can inhibit the activity of NF-κB by suppressing the phosphorylation-dependent proteolysis of Iκ-B-α in LPS-stimulated RAW 264.7 cells.

In addition, (±)-DHIE suppresses TNFα production in DNP-HAS-stimulated RBL-2H3 cells [70]. Similarly, (+)-DHIE inhibits the release of TNF-α in antigen-stimulated RBL-2H cells [71]. Altogether, these data suggest that both the (±)-DHIE and (+)-DHIE decrease the production of the pro-inflammatory mediators. Taken together, these data suggest that DHIE could act as an anti-inflammatory agent through the inhibition of NF-κB activation.

### 6.6. Other Biological Properties of DHIE

Takeyoshi et al. evaluated the skin sensitization potencies induced by DHIE. In this sense, DHIE was classified as a moderate sensitizer and showed a 50% of sensitization rate in the guinea pig maximization test and was classified as a moderate sensitizer in the non-RI LLNA test, this compound induced dose-depend on lymph node cell proliferation, and its E3 value was estimated as 9.4% [72].

In addition, it has been analyzed the interaction between DHIE and peroxisome proliferator-active receptor (PPAR), which is a receptor target for the treatment of diabetes mellitus type 2. In this line, DHIE was shown to act as an agonist on the PPAR ligand binding domain [73,74]. Muchrtaridi et al. demonstrated that DHIE formed a hydrogen bond network of His323, Tyr379, His449, and Ser489. The hydrophobic tail of the DHIE fitted into a “diphenyl pocket”. Thus, DHIE might be potent as an agonist PPAR [74].

The adipocyte browning and its associated metabolic have favored the search for natural compounds that can be able to treat metabolic diseases. In this line, Yoon et al. demonstrated that DHIE induced the expression of the proteins characteristic of brown-like adipocytes in C3H10T1/2 mesenchymal stem cells. DHIE induced uncoupling protein 1 (Ucp1) and expression of other thermogenic genes in C3H10T1/2 mesenchymal stem cells via a mechanism involving protein kinase A (PKA). DHIE treatment also inhibited the expression of white-adipocyte–specific genes. Moreover, DHIE treatment promoted lipolysis via PKA mediated pathway [75]. Taken together, DHIE is an inducer of brown-like adipocyte formation with lipolytic properties, suggesting that DHIE could be used as a potential anti-obesity agent. However, it is necessary to realize studies in both animal models and clinical ones that support this hypothesis.

El-Alfy et al. studied whether DHIE could indirectly interact with the endocannabinoid system via inhibiting the fatty acid amide hydrolase (FAAH) and monoacylglycerol lipase (MAGL) enzymes. In this context. DHIE was able to inhibit FAAH with an IC_50_ of 7.02 μM but did not exhibit MAGL inhibition [76]. These data suggest that DHIE, by inhibiting FAAH could induce its biological effects through the endocannabinoid system. However, it is necessary to carry out in vivo studies to confirm these findings.

## 7. Pharmacokinetic of DHIE

Pharmacokinetics studies the dynamic movements of chemical compounds during their passage through the body and, as such, encompasses the kinetics of absorption, distribution, metabolism, and excretion (ADME). Proper characterization and understanding of the pharmacokinetic properties of a new compound are critical for safe and effective drug development.

**Figure 5:**
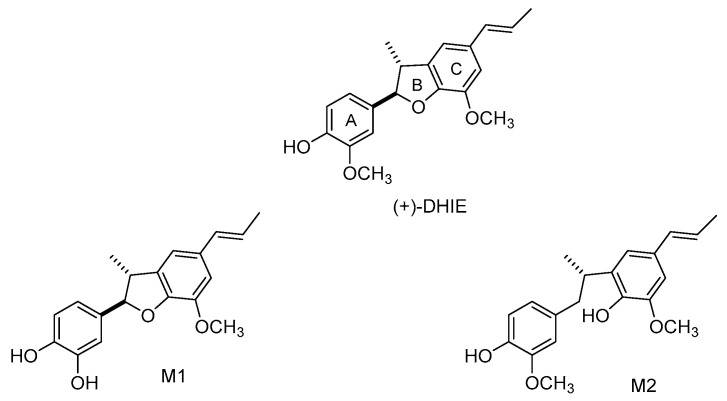
Metabolites of DHIE.

A study demonstrated that after 16 min of administration of DHIE (100 mg/kg, intravenous) to rats, the highest concentration of this compound was detected in the liver, lung, kidneys, spleen, heart, muscle, testes, stomach, intestine, and brain [77]. The principal tissues where DHIE remained for a longer time were the liver, brain, and intestine. These observations suggest that DHIE is stable and widely distributed in the body. In consequence, the liver, brain, and intestine are likely to be the most important target tissues involved in the biological effects of DHIE. A previous study demonstrated that DHIE showed high concentration in cerebral nuclei such as the hippocampus, striatum, cortex, cerebellum, brainstem, and hypothalamus at 8 min after the administration of the compound [78]. In addition, a study demonstrated that the permeability of DHIE is moderate as its apparent permeability (Papp) value is about 10^−6^ cm/s, and the primary transport mechanism for DHIE was passive diffusion [79]. These observations suggest that DHIE could cross the blood-brain barrier (BBB) rapidly and could be useful for the treatment of diseases that involve the central nervous system. However, further studies are necessary to corroborate this hypothesis. In addition, DHIE showed a distribution half-life (t_1/2α_ = 26.8 ± 0.4 min) and an elimination half-life (t_1/2β_ = 389.1 ± 76.3 min), and the volume of distribution in the central compartment (Vc) of DHIE was 0.197 ± 0.003 mg/(μg/mL) [80]. These data suggest that DHIE is quickly absorbed and well distributed throughout the body. Regarding metabolism, DHIE undergoes demethylation and a ring-opening reaction in vivo after administration to rats [81]; the main metabolites are M1 and M2 (Figure 5). Finally, the excretion of DHIE in urine and faeces was studied after intravenous and intragastric administration to rats. The amount of DHIE and its metabolites excreted was higher in faeces than in urine, suggesting that DHIE and its metabolites are eliminated principally in the faeces [82].

## 8. Biological Activity of Semi-Synthetic DHIE Derivatives

Compound **1** has been evaluated in vitro for trypanocidal activity against trypamastigote forms of *T. cruzi*. This compound displayed parasite lysis of 21.1% and IC_50_ values of 28 µM [18], and 378.4 µM [52]. In contrast, DHIE was more active as parasite lysis was greater than 50% with the IC_50_ = 100.8 µM value [52].

In Addition, compound **1** showed a cytotoxic activity of 52.8% for HCT-166 (human colorectal carcinoma), 52% for MCF-7 (human breast adenocarcinoma) and 13.9% for K562 (chronic myeloid leukemia), did not show activity for human cell line HL-60 (acute promyelocytic leukemia) [51].

Compound **2** showed a minimum cytotoxic activity on cell lines MCF-7 (0.98%) and HL-60 (9.47%). Moreover, this compound did not exhibit cytotoxic activity in K562 and HCT-166 cell lines. Compound **3** did not show cytotoxic activity for HCT-166, MCF-7, K562 and HL-60. However, compound **4** exhibited a high cytotoxic activity on the HL-60 (96.9%), MCF-7 (18.7%) and K562 (16.8%) and showed no effect on tumor cell HCT-166 viability. Moreover, compound **4** was also cytotoxic to human non-tumor cells (PBMC), with a 66.9% of inhibition [51].

Compounds **5** and **6** were evaluated against trypomastigote forms of *T. cruzi*, showing the IC_50_ of 450.7 and 399.0 µM values, respectively [52]. Comparing DHIE with compounds **2**, **5**, and **6**, the presence of a hydroxyl group in DHIE enhances trypanocidal activity.

Alvarenga et al. demonstrated the antimycobacterial ability of the compounds **5**, **7**, **8**, **9**, **10**, **11** and **12** (Figure 4) against *M. massiliense*, *M. fortuitum* and *M. abcessus*, as well as the inhibition effect of biofilm formation fast-growing mycobacteria. This study showed that compounds **11** and **12** exhibited a significant inhibitory effect on planktonic growth of the three stains of mycobacteria tested, with even lower MIC values than those observed with DHIE. They showed that compounds **11** and **12** were more effective in inhibiting microbial film formation than DHIE [54]. Therefore, it appears that the presence of an alcoholic hydroxyl group may favor the interaction with receptors to be able to induce susceptibility of the three stains of mycobacteria tested.

The semi-synthetic derivatives **1**, **6**, **13**, **14** and **15** were tested against *T. cruzi*. The antitrypanosomal activity for all compounds tested was higher than DHIE. Nevertheless, these compounds exhibited activity only against the trypomastigote form and were not active against the intracellular amastigote. Compound 14 exhibited higher activity against trypomastigotes of *T. cruzi* (IC_50_ = 5.0 µM), followed by compound **15**, showing IC_50_ = 10.5 µM and compound **6**, exhibiting IC_50_ = 17.9 µM. These data suggest that the presence of an additional substitution in the aromatic ring of DHIE contributed to the antitrypanosomal activity. However, this compound presents limited oral bioavailability estimation (<85%, Paap < 1.0 × 10^−6^ cm/s) in parallel artificial membrane permeability assays (PAMPA) due to excessive lipophilicity [18].

Compounds **17a**–**d**, as well as **18a**–**d**, were evaluated in vitro to α-glucosidase. The compounds **17a**–**d** were inactive against α-glucosidase; however, compounds **18a** exhibited an IC_50_ = 0.23 mmol/L, **18b** IC_50_ = 0.33 mmol/L, **18c** IC_50_ = 0.36 mmol/L and **18d** the IC_50_ = 0.38 mmol/L against α-glucosidase, were less compared to acarbose with an IC_50_ = 0.054 mmol/L [36]. All these compounds showed a moderate inhibitory activity to α-glucosidase close to acarbose.

## 9. Conclusions

This review shows that DHIE can be obtained in good yields from isoeugenol using metallic catalysts or enzymes. In this regard, HRP is the best option to obtain (±)-DHIE; coconut water is better for obtaining only the (−)-DHIE enantiomer. Besides, DHIE has moderated sensitivity and low toxicity. DHIE exhibits a broad spectrum of biological activities, such as anti-oxidant, anti-inflammatory, anti-parasitic, anti-microbial, especially against some *Mycobacterium* species, and cytotoxic against different cancer cell lines. Also, DHIE produces anti-diabetic and anti-obesity activity. These data indicate that DHIE could be used to treat diseases, especially those caused by parasites such as *T. cruzi* and *S. mansoni*, because there are few drugs that can be used against them, and they have low effectiveness. Moreover, DHIE shows a good distribution and crosses the brain barrier suggesting that it can be used in nervous system diseases. Finally, the semi-synthetic derivatives from DHIE were obtained from a simple chemical modification of the DHIE in propenyl chain and phenolic hydroxyl, improving the biological effect and pharmacokinetic properties.

## 10. Perspectives

Recently, there has been a particular interest in DHIE properties. DHIE provide effect against a broad spectrum of bacterial, parasitic, and cytotoxic against different cancer cell lines. Moreover, DHIE shows anti-oxidant and anti-inflammatory effects. However, more systematics preclinical work and clinical trials are necessary before it can be considered as a potential drug to improve health in either acute or chronic diseases such as anti-bacterial, anti-parasitic, anti-inflammatory, anti-diabetic, and anti-obesity.

## Figures and Tables

**Table 1:** Natural sources of DHIE.

Plant	Part	Ref
*Aristolochia taliscana*	Roots	[13]
*Aristolochia elegans*	Wild roots	[14]
*Nectandra glabrescens*	Fruits	[15]
*Nectandra rigida*	Bark and stems	[16]
*Nectandra oppositifolia*	Leaves	[17,18]
*Machilus thunbergii*	Bark	[19]
*Myristica fragrans*	Aril	[20,21]
*Eupomatia laurina*	Bark	[22]
*Magnolia kachirachirai*	Leaves	[23]
*Piper kadsura*	Aerial parts	[24]
*Piper wallichii*	Aerial parts	[25]
*Piper capense*	Fruits	[26]
*Licaria aritu*	Wood	[8]
*Machilius odoratissima*	Bark	[27]
*Machilus obovatifolia*	Root	[28]
*Magnolia ovata*	Unripe fruits Leaves	[29] [30]
*Saururus chinensis*	Aerial parts	[31]
*Urbanodendron verrucosum*	Branch wood	[32]
*Nardostachys chinensis*	Roots and rhizomes	[33]

**Table 2:** Condition for radical dimerization of IE for obtained DHIE.

Catalyst	Mol-Ratio	Solvent	T (°C)	Reaction Time (h)	Yield (%)	Reference
FeCl_3_	1.4	water-ethanol	4	24	30	[34]
FeCl_3_	0.5	water-ethanol	5	24	22	[35]
Ag_2_O	0.5	toluene-acetone	−70	48	40	[36]
CAN	1.5	tetrahydrofuran	room temp	0.5	81	[37]
IDA	0.62	dichloromethane	room temp	48	35	[38]

**Table 3:** Synthesis of DHIE from IE with HPR.

IE (mmol)	Methanol/Buffer (%)	U	H_2_O_2_ (mmol)	T (h)	DHIE	Yield (%)	Reference
6	26:74 Phosphoric pH = 6.0	4.5–7.5 × 10^−6^ mmol	0.5	24	NR	22	[40]
4.9–10	10:90 Citrate-phosphate pH = 3.0	2150–4300	2.45–5	1	(±)	60	[39]
10	10:90 Citrate-phosphate pH = 3.0	2500	5	1	(±)	99	[41]
6.2	10:90 Citrate-phosphate pH = 3.0	1500	3.05	0.33	(±)	27	[42]

NR = not reported.

## Data Availability

Not applicable.
